# Registration of carpometacarpal arthroplasty in the Dutch arthroplasty register: Impacting factors and participation

**DOI:** 10.1016/j.jpra.2025.02.004

**Published:** 2025-02-06

**Authors:** Antonius A. van den Hurk, Thomas M.A.S. Lauwers, Anneke Spekenbrink-Spooren, Juliëtte E. Hommes, René R.W.J. van der Hulst, Rutger M. Schols, Xavier H.A. Keuter

**Affiliations:** a Department of Plastic, Reconstructive, and Hand Surgery, Maastricht University Medical Center+, Maastricht, The Netherlands; b Dutch Arthroplasty Register (LROI), ’s-Hertogenbosch, The Netherlands; c Department of Plastic, Reconstructive, and Hand Surgery, Haaglanden Medical Center, The Hague, The Netherlands; d Department of Plastic, Reconstructive and Hand Surgery, Zuyderland Medical Center, Sittard-Geleen/ Heerlen, The Netherlands; e Department of Plastic, Reconstructive and Aesthetic Surgery, University Hospital Brussels, Free University Brussels (VUB), Brussels, Belgium

**Keywords:** Carpal arthroplasty, CMC-1 arthroplasty, Registry study, Orthopedic & plastic surgery, Observational early outcomes

## Abstract

The treatment of carpal- and carpometacarpal arthritis using arthroplasty is becoming more common in hand surgery. Trapeziectomy is regarded as the standard treatment. Complications of trapeziectomy like thumb shortening and reduced mobility may be less common in arthroplasty. However, arthroplasty may cause implant failure and possible revision surgery. The Dutch arthroplasty registry (LROI) has been registering hand and wrist implants since 2016.

Data from the LROI from 2017 to 2022 was used aiming to assess descriptive data regarding carpal- and carpometacarpal arthroplasty to demonstrate associations between arthroplasty and possible risk factors. Furthermore, this study aims to demonstrate the potential of the LROI and assess registration completeness.

The registry included 178 primary first carpometacarpal arthroplasties, along with 23 revision arthroplasties. About 69.1 % of primary surgeries was performed by plastic surgeons, the others by orthopedic surgeons. Primary surgery was performed in women in 74.2 % of cases. Revision arthroplasty was performed as often by plastic surgeons, as by orthopedic surgeons. Too few carpal implants were registered to assess these types of implants.

Comparing the registrations in the LROI with the national healthcare claims database showed a completeness of 9.04 % for plastic surgeons, and 30.39 % for orthopedic surgeons. This low registration completeness did not allow for any definitive conclusions to be drawn. However, this study shows large-scale registries may provide useful insights, possibly guiding clinical decision-making. To improve registration completeness, efforts should be made to facilitate registration as quick as possible, while also boosting awareness among physicians that perform carpal- and carpometacarpal arthroplasty.

## Introduction

Osteoarthritis, osteonecrosis, or other problems of the carpal bones can cause significant pain and loss of mobility in patients. Carpal disease may involve multiple carpal bones and can sometimes require complex surgery. Patients with severe complaints of the carpal bones, who do not respond to conservative treatment, usually undergo carpal resection surgery or arthrodesis of the affected joint. Surgical intervention usually includes resection of carpal bones and thereby alters the carpal anatomy and biomechanics. Theoretically, to preserve the original biomechanics of a joint, an implant may be beneficial as it keeps the trapezium in place while treating the arthritis. This is why the concept of using joint implants has become more popular in the last decades.

The most common problem regarding the carpal bones is osteoarthritis of the thumb carpometacarpal joint (CMC-1). In the current treatment of CMC-1 arthritis, trapeziectomy is the most common procedure and is therefore considered standard care in The Netherlands by most surgeons. The trapeziectomy can be combined with ligament reconstruction and tendon interposition (LRTI) with multiple techniques described in the literature to improve stability in the resection cavity.^1^ Possible complications of trapeziectomy with or without LRTI include thumb shortening, reduced mobility, loss of grip strength, and a long duration of recovery.^2^, ^3^, ^4^ The Dutch guideline for treatment of thumb osteoarthritis was most recently updated in 2015 in which it recommended trapeziectomy without additional ligament reconstruction for surgical treatment of isolated CMC-1 arthritis.^5^ Promising techniques that have gained more attention since the updated guideline include denervation of the joint^6^ as well as arthroplasty using implants to reconstruct the joint as close to its anatomical function as possible by not removing a carpal bone.

CMC-1 arthroplasty using implants has been reported to preserve mobility, reduce pain, treat possible Z deformity and maintain or improve thumb height thereby improving grip strength.^7^, ^8^, ^9^ However, the superior surgical treatment for CMC-1 is a subject drawing much discussion. The concept for this technique was first introduced by Swanson in 1968.^10^ Since then, implants have evolved in composition, shape, and surgical implantation. Efforts were made to preserve the original saddle-like shape of the trapezium. However, due to complication rates, a ball-and-socket design was deemed the superior concept.^3^^,^^11^ Implants therefore include both a carpal and metacarpal part which can be made from different materials. Dislocations of CMC-1 implants remained a major problem. Recently the concept of dual mobility implant has gained popularity, as these implants have shown improved stability with less dislocations.^12^^,^^13^ Globally, this procedure is performed with ever evolving implants, of which multiple generations currently exist. As the Netherlands use a strong “evidence based” reimbursement system of health care, arthroplasty of the CMC-1 joint is still less common and solely recommended in research settings due to a relatively small evidence base.^5^

Arthroplasty surgeries in other carpal joints are less common than CMC-1 joint arthroplasty. Indications could be osteoarthritis of the carpal joints, non-union after carpal fracture or avascular necrosis. Implants have also been developed for several carpal bones to perform arthroplasty surgery, for example the lunate,^14^ the scaphoid,^15^ and carpectomy of the proximal row combined with a pyrocarbon capitate resurfacing implant.^16^ These implants are infrequently used since indications are rare.

The Dutch Arthroplasty Registry LROI, known in Dutch as ‘Landelijk Registratiepunt Orthopedische Interventies’ started their registry in 2016 and is only the second registry globally which registers hand and wrist implants separately and therefore enables specific research on carpal arthroplasty. The only other registry which also specifically registers carpal implants is the Norwegian registry. The data from this registry describes a 5-year survival rate of 90–94 % of CMC-1 implants used for arthroplasty. They found that gender, age, and diagnosis had no significant impact on the risk of revision.^17^ To our knowledge, no further studies were conducted reviewing the registration and performance of carpal implants.

The aim of this study is to research and analyze descriptive data (patients demographics, surgeon specialty, indications for arthroplasty) regarding carpal- and carpometacarpal arthroplasty from 2017 to 2021 with the goal to demonstrate associations between arthroplasty and possible risk factors. Furthermore, this study aims to demonstrate the potential of the arthroplasty registry (LROI) for clinical and research purposes and define current registration completeness.

## Methods

### Study design

The current study is based on data provided by the LROI registry. Data was extracted from the LROI database concerning demographics, patient characteristics, clinical characteristics, operative details, and revision details. This data was analyzed and reported in this study.

### Participants

Patients were included through registry of their respective surgeries in the LROI database. Patients were included in the database if they were 18 years of age or older and had a primary or revision arthroplasty of the carpometacarpal joint between 2017 and 2022 which was registered in the LROI database by the surgeon performing the operation. The originally proposed design included data gathered on all available carpal and carpometacarpal implants. However, data on implants other than CMC-1 was scarce. Therefore, this study only focusses on implants in the first carpometacarpal joint. Moreover, total- or hemi-wrist arthroplasties were also excluded since these do not involve arthroplasty of the carpal joints. Patients were treated in different medical centers across The Netherlands, no specific information on the medical centers was provided.

### Data management

The data was obtained from the LROI in a secure file transfer and was stored on the encrypted drive of the department of plastic and reconstructive surgery in the Maastricht University Medical Centre (MUMC+). The LROI provided two different databases. The first database contained the primary arthroplasties, the revisions of the respective primary surgeries were also registered. The second database included all revision arthroplasties regardless of the presence of registration of the primary surgery in the LROI database.

### Assessment of registration completeness

To assess the completeness of registration we used the national healthcare claims database. This database is used by the healthcare insurance to register all provided healthcare and reimburse the relevant costs. The claims database uses healthcare activities for reporting. We identified all healthcare activities that mentioned carpometacarpal arthroplasty. We found two healthcare activities: 038310 (‘implantation of a carpal implant’) and 038315 (‘removal of a carpal implant). We then accessed the healthcare claims database and counted all registrations of these healthcare activities between 2017 and 2022. This provided an estimate which we compared with the number of registrations in the LROI database. This method was used in earlier research into finger arthroplasty and validated when compared to the Dutch breast implant registry (DBIR).^18^^,^^19^

### Statistical analyses

The current study uses descriptive statistics to describe patient characteristics, operative details, implant details, and revisions. More advances statistical analyses were not justified based on the low registration completeness. IBM SPSS 28 was used for the statistical analyses.

## Results

### Primary surgery

The database with primary carpometacarpal implants comprised 178 patients. Of these patients, the majority was female (74.2 %). The mean age in our study population was 60.26 years with a standard deviation of 7.71 years. Patients had an average BMI of 27.28 with a standard deviation of 4.20 kg/m^2^. Most patients were classified as ASA class II (58.4 %), followed by ASA I (32.6 %). The highest number of operations was performed in the left hand at 57.9 %. No operations used cement, although eleven operations did not report information on this aspect. Most arthroplasties were approached dorsally. Plastic surgeons performed 69.1 % of all operations, the remaining surgeries were performed by orthopedic surgeons. Three revisions were registered with their respective primary surgery also registered. The revised implants included one ARPE and one NuGrip implant, the third implants was not specified. The reason for revision was not specified in the NuGrip implant, the APRE was revised due to dislocation. See Table 1 for an overview.

**Table 1:** Patient- and clinical characteristics in primary carpometacarpal arthroplasty.

Characteristic	*n* = 178	Missing
Gender (%)		0
- Male	46 (25.8)	
- Female	132 (74.2)	
Age; Mean ± SD, years	60.26 ± 7.71	
BMI; Mean ± SD, kg/m²	27.28 ± 4.20	7 (3.9)
Smoking (%)		6 (3.4)
- Yes	28 (15.7)	
- No	144 (80.9)	
ASA classification (%)		1 (0.6)
- I	58 (32.6)	
- II	104 (58.4)	
- III-IV	15 (8.4)	
Side (%)		0
- Left	103 (57.9)	
- Right	75 (42.1)	
Cemented (%)	0	11 (6.2)
Approach (%)		3 (1.7)
- Dorsal	140 (78.7)	
- Volar	14 (7.9)	
- Lateral	9 (5.1)	
- Other	12 (6.7)	
Specialism surgeon (%)		5 (2.8)
- Plastic	123 (69.1)	
- Orthopedic	50 (28.1)	
Revision (%)	3 (1.7)	0

### Revisions

In the LROI, 23 revisions were registered. Most of these revisions did not have a primary surgery registered (*n* = 20). Most of the patients were female at 69.9 %. The mean age was 61.04 with a standard deviation of 7.88 years. The mean BMI was 27.24 with a standard deviation of 4.07. Three patients were smokers. The left hand was slightly more commonly revised (*n* = 13). One revision used cement in the procedure. Revisions were performed equally as much by plastic surgeons and orthopedic surgeons. The revisions without a primary operation registered did not specify the removed implant. For two revisions, a re-revision was necessary. Unfortunately, the explanted implant was not specified and could therefore not be identified. See Table 2 for an overview.

**Table 2:** Patient- and clinical characteristics in CMC-1 revision arthroplasty.

Characteristic	*n* = 23	Missing
Gender (%)		0
- Male	7 (30.4)	
- Female	16 (69.9)	
Age; Mean ± SD, years	61.04 ± 7.88	
BMI; Mean ± SD, kg/m²	27.24 ± 4.07	1 (4.3)
Smoking (%)		0
- Yes	3 (13.0)	
- No	20 (87.0)	
Side (%)		0
- Left	13 (56.5)	
- Right	10 (43.5)	
Cemented (%)	1 (4.3)	1 (4.3)
Specialism surgeon (%)		0
- Plastic	11 (47.8)	
- Orthopedic	12 (52.2)	
*Re*-revision (%)	2 (8.7)	0

### Carpal implants

As stated, carpal implants were not the within the scope of this study. Mainly because the data on these implants was very limited. Table 3 shows the number of carpal implants registered. The majority were pyrocarbon capitate resurfacing implants (PRCI) comprising 74.2 %. Next were Amandys implants comprising 20.0 %. Furthermore, a single adaptive proximal scaphoid implant and a lunate implant were registered.

**Table 3:** Number of carpal implants registered in the LROI.

Implant	Number (*n* = 35)
PCRI	26 (74.2 %)
Amandys	7 (20.0 %)
APSI	1 (2.9 %)
Lunate	1 (2.9 %)

PCRI, Pyrocarbon capitate resurfacing implant; APSI, Adaptive proximal scaphoid implant.

### Registration completeness

In the national healthcare claims database (NHCD), a total of 1685 operations involving a carpometacarpal implant have been registered in the national healthcare insurance database. Of these 1685 operations, 1345 were primary implantations while 340 were removals or revisions. Plastic surgeons performed 1481 of these operations, while orthopedic surgeons performed 204 operations. In the LROI database, significantly less operations were registered. A total of 201 carpometacarpal implants were registered, consisting of 178 primary operations and 23 revisions. Thereby, the registration completeness appeared to be 9.04 % for plastic surgeons and 30.39 % for orthopedic surgeons (Table 4).

**Table 4:** Registration completeness of CMC-1 arthroplasty in the LROI.

	Operations registered in the NHCD	Operations registered in the LROI	Registration completeness
Plastic surgeons	1481	134	9.04 %
Orthopedic surgeons	204	62	30.39 %

## Discussion

This study aimed to identify impacting factors for carpometacarpal implant performance, based on data from the Dutch Arthroplasty Registry LROI, representing the concept of big data. It is the first study describing the outcomes of the national database in The Netherlands for carpometacarpal implants. The number of implants registered in the database did not justify any statistical analyses other than descriptive statistics. However, trends can be observed in the small sample that was registered.

For example, we see most implants being used in female patients. This is in line with the general understanding that arthritis of the first carpometacarpal joint is most common in female patients.^20^ Moreover, the Norwegian arthroplasty registry found similar numbers regarding the gender of patients.^17^ In the revision database, we found the ratio to be roughly the same. Indicating that, while the prevalence of primary implantations is higher in women, the revision rate may be similar between the sexes. This is consistent with findings of the Norwegian registry.^17^

Primary operations and revisions were more common in the left hand. The difference was relatively equal in primary surgery and revisions, indicating the left hand as being more commonly operated on primarily. This is notable since carpometacarpal arthritis is usually associated with physical strain on the joint. Since most of the population is right-handed, we expected the prevalence of primary and revision operations to be higher in the right hand. A theory states that the non-dominant hand is more commonly engaged in more static activities and therefore lack muscle and ligament development which makes them more susceptible to stress.^21^ The prevalence of the left hand may also be due to a smaller sample size.

The literature on carpal arthroplasty is not yet extensive. Recently, a large scale RCT between trapeziectomy and CMC-1 joint arthroplasty by De Jong et al. was published describing the absence of a significant difference in MHQ scores. However, outcomes regarding strength, range of motion, and early patient satisfaction were deemed significantly higher in the arthroplasty group.^9^ Furthermore, significantly better function is described for CMC-1 arthroplasty compared to trapeziectomy by Raj et al.^22^ Moreover, they described higher probabilities of complications and revision surgery in the arthroplasty group.^22^ Registration using national databases could facilitate easier and more complete comparison between the current standard of care and arthroplasty.

One of the main outcomes that registry data would provide is information on the survival and compatibility of different implants. This way, we might use big data to influence clinical decision-making. In the current database, 178 primary operations are registered. In this group, three implants have been revised at least once, providing a revision rate of 1.7 %. The ARPE implant and the NuGrip implant were both revised at least once. Other revisions did not have a primary surgery registered. Therefore, no information exists on the primary implant. Both ARPE and NuGrip implants are not part of the newest generation. However, to draw any conclusions on revision rates and implant survival, the data had to be representative and complete. Currently the registration completeness is not sufficiently high enough to allow these conclusions to be drawn.

The registration completeness is lower for plastic surgeons than for orthopedic surgeons. We have observed this discrepancy before in earlier research on finger implants in the LROI^18^ It is known that orthopedic surgeons are more strongly monitored in the registration of hand implants than plastic surgeons. Moreover, the LROI is more commonly known in the orthopedic world since it is also used for hip-, knee-, shoulder, and other arthroplasties. In plastic surgery it is only used for hand arthroplasty. However, in our previous research, the registration completeness for the orthopedic surgeons was much higher than in carpometacarpal implants. The extremely low registration completeness in carpometacarpal implants may be a result of the less adopted nature of this treatment in The Netherlands. Therefore, implants may be registered in smaller research databases and not in the national database. Next, due to the absence of a financial compensation for the implant, the urge to register it correctly may not be as prominent, leading to inconsistencies in registration. To encourage registration completeness and access the highest potential of completeness, mandatory registration seems to be the most effective way. Especially when looking at the Dutch Breast Implant Registry (DBIR) in which registration completeness of >95 is achieved.

At the onset of the research involved in this paper, we aimed to investigate all carpal implants as opposed to only first carpometacarpal implants. As described earlier, this was not possible due to the low number of cases registered. Multiple pilot studies have been conducted into a range of different implants. A study investigating lunate implants reported loss of mobility but increase of grip strength and decrease of pain.^14^ Total scaphoid implants have also been described for problems like non-union of the scaphoid after fracture injury. In these cases, restoration of function and decrease of pain were achieved.^15^ Proximal row carpectomy combined with pyrocarbon capitate resurfacing implant has been described for severe radiocarpal osteoarthritis and osteoarthritis of the proximal pole of the capitatum. Satisfactory results were described, however no significant difference with only carpectomy was found.^16^ A registry could provide large scale databases which would not be possible otherwise considering the uncommon nature of carpal arthroplasty.

This study has two main limitations. Firstly, and most important, the registration completeness was not optimal. This prevented us from performing robust statistical analyses and limited the results to descriptive statistics. This limitation also underlines the main take-away from this research, namely the need to register more thoroughly. Secondly, the database only encompasses the Dutch healthcare system. In The Netherlands, carpometacarpal arthroplasty is not yet widely adopted. Therefore, the population in which the treatment is applied may not be representative of the general population with carpometacarpal arthritis.

Based on the findings from this research, surgeons should be encouraged to strive for better registration completeness, as this could provide much needed clarity and uniformity in the field of carpometacarpal arthroplasty. The potential for big data is obvious, especially in making informed clinical decisions, and we have the tools in place to unlock this potential. It would be worthwhile exploring options to make registration less tedious or time-consuming. Moreover, centralizing the databases and make them uniform across Europe or even globally would be beneficial to easily analyze big data. Further research should focus on obtaining data from large-scale registries, or on optimizing the participation of surgeons in such registries. Efforts could focus on improving the registration process or creating awareness for registries in the surgical field.

## Conclusion

Registration completeness is too low to draw any conclusions on implant performance, although revision rates seem low. Based on this limited database, women are more likely to undergo first carpometacarpal joint arthroplasty. The main take-away of this study should be the need for complete implant registration in carpal arthroplasty to enable evidence based clinical decision making using large and representative databases.

## Funding

The authors received no financial support for the research, authorship, and/or publication of this article.

## Ethical approval

Not applicable.

## Informed consent

Not applicable.

## Author contribution

AS delivered the data from the LROI database, AvdH analyzed data obtained from the LROI and wrote the first draft of the manuscript. All authors reviewed and edited the manuscript and approved the final version of the manuscript.

## Declaration of competing interest

AS is affiliated with the LROI as a researcher. The remaining authors do not have any conflicting interests.
